# Students’ Feedback about Feedback; Have our PBL tutors started the shift towards a dialogic ask-tell-ask approach?

**DOI:** 10.12669/pjms.36.7.1778

**Published:** 2020

**Authors:** Majda Saeed, Arthur C. Isnani, Samina A. Khan, Nehal Khamis

**Affiliations:** 1 Majda Saeed, Medical Education Department, College of Medicine, King Saud University, Riyadh, Saudi Arabia; 2 Arthur C. Isnani, Obesity Research Center, College of Medicine, King Saud University, Riyadh, Saudi Arabia; 3 Samina A. Khan, Medical Education Department, College of Medicine, King Saud University, Riyadh, Saudi Arabia. National Advanced IPv6 Centre (NAv6), Universiti Sains Malaysia, Pulau Penang, Malaysia; 4 Nehal Khamis, Saudi Commission for Health Specialties, Riyadh, Saudi Arabia. Pathology and Medical Education Departments, College of Medicine, Suez Canal University, Ismailia, Egypt

**Keywords:** Problem-based learning feedback, ASK-Tell-ASK, bi-directional, Dialogic, Perceptions

## Abstract

**Objective:**

A paradigm shift towards a PBL bidirectional dialogic feedback can enhance learners’ performance. This study aimed to investigate undergraduate medical students’ perceptions of their PBL feedback.

**Methods:**

We sent e-mail invitations to a web-based survey to year one and two students at College of Medicine, King Saud University. Items included the process, content, and benefits of PBL feedback.

**Results:**

Of 209 respondents, 110 (53%) were first and 99 (47%) were second-year students. About 50% agreed that the feedback was regularly provided at scheduled timing and 72% perceived feedback environment as non-threatening. Agreement rates that the tutors asked students first to assess their performance, tell them what went well, what the areas for improvement are and develop with them an improvement plan were 59%, 61%, 61% and 52%, respectively. 61% agreed that tutors judged performance not personality. More year one students significantly agreed that the PBL feedback helped them to improve their knowledge acquisition and non-technical skills.

**Conclusion:**

Many of our PBL tutors have started the shift to a dialogic bi-directional feedback. We recommend continuing the faculty development efforts, peer-reviewing, and seeking student’s feedback within the academic quality satisfaction surveys.

**Abbreviations:**
PBL: Problem-based learning
ATA: Ask-Tell-Ask
CoM: College of Medicine
KSU: King Saud University

## INTRODUCTION

The recent approaches to effective feedback show a paradigm shift from the traditional, unidirectional, teacher-focused “provided” feedback to a bidirectional “dialogic” one. The emphasis is on eliciting a learner’s behavior change through establishing a teacher-learner conversational relationship.^1^,^2^ Clear feed-forward goals are key to performance improvement in future tasks.^3^

In the conversational “Ask-Tell-Ask” (ATA) feedback approach: “Ask” means to ask the learners to assess their performance, “Tell” means sharing teacher’s impressions of positive behaviors and areas for improvement. The second “Ask” is about checking the learners’ understanding and allowing them to develop with the teacher a plan for future improvement.^4^

At the College of Medicine of King Saud University (COM, KSU), Riyadh, Saudi Arabia, a hybrid PBL curriculum was implemented in the academic year 2009/2010. Two students’ feedbacks are planned by the PBL unit, an individualized one at mid-block, and another within-the-group one by the end of the second PBL tutorial. As per the College’s structured form, the feedback content should cover knowledge, cognitive skills, interaction, and contribution to group function. All our PBL tutors are required to attend a faculty development workshop that prepares them to become PBL facilitators. Provision of effective, conversational feedback is also tackled in the faculty development unit’s workshops.

This study aimed to investigate our undergraduate medical students’ perceptions of the process and value of PBL feedback. It tries to answer the following question from students’ perspective: Have our PBL tutors started the shift towards a bidirectional conversational feedback approach that targets the continuous improvement of learner performance? To our knowledge, this is the first study to investigate the process, compliance with the current trends and impact of PBL feedback regionally.

## METHODS

This was a cross-sectional survey study. After an orientation session, group leaders sent e-mail invitations with the survey link to the 616 first and second-year medical students enrolled at COM, KSU in the academic year 2015-2016. Students were assured that participation is voluntary and responses will be anonymous. The COM, KSU Research Ethical Committee approved the study (Ref.No# 14/4271/IRB, Date: May 21, 2014, Renewal Ref. No. 20/0704/IRB Sept. 27, 2020). We sent two reminder emails after one and two weeks.

After reviewing the relevant literature, two of the authors (MS and NK) developed the survey using survey monkey (http://www.surveymonkey.com/). It was then reviewed by two medical education experts for content validity. We piloted the survey among ten students before the actual data collection. The domains investigated included (A) Feedback process: frequency and timing, environment, ATA steps, tutor performance, and content. (B) Feedback’s helpfulness in improving: knowledge acquisition, communication, problem-solving, teamwork, and self-assessment skills, and (C) Perceived value of the feedback. Agreement responses were rated on a five-point Likert scale ranging from one (strongly disagree) to five (strongly agree).

### Statistical Analysis:

The collected data was analyzed using the Statistical Package for Social Sciences (SPSS) version 23.0 (SPSS Inc., IBM, Armonk, New York, USA). Descriptive analysis was reported as frequencies, mean values, and standard deviation. Overall scores for students’ rating were reported as means and standard deviations. Chi-square test (*x*^2^) was used to determine statistically significant differences between responses’ percentages according to year levels. A p value of ≤0.05 was considered statistically significant.

## RESULTS

### Response Rate

Two hundred and nine students responded (209/616, 34%); 110 (53%) first-year and 99 (47%) second-year students. Seventy-three (35%) of all respondents were males, and 136 (65%) were females.

### 1. Process of the PBL feedback:

- ***Frequency and timing:*** Ninety five respondents (46%) strongly agreed/agreed on regularly receiving an individual mid-block feedback (Mean of 3.43 ± 1.13). Also, 49% (102/209) agreed/strongly agreed that there was a protected time for individual feedback within the group at the end of the 2^nd^ session of each PBL case (Mean of 3.40 ± 1.10
- ***Environment:*** One hundred fifty-one students (72%) agreed/strongly agreed that the feedback sessions were conducted in a relaxed and non-threatening environment (Mean of 3.91 ± 1.09).
  There were no statistically significant differences regarding feedback frequency, timing and environment across year levels (p>0.05).
- ***Steps of the PBL Feedback Process:*** The students mean rating for the feedback steps was positive (3.56 to 3.67/5) (Table-I). Regarding the dialogic ATA feedback model; 124 students (59%) agreed/ strongly agreed that the tutors ask them to assess their own performance as a first step, 128 (61%) agreed/strongly agreed that they then tell them what went well, 127 (61%) students agreed/strongly agreed that tutors follow this by telling them areas for improvement, and 108 (52%) agreed/strongly agreed that tutors develop with them a plan for improvement. An average of 26% were not sure if the first three steps were regularly followed by their tutors. Eighty-five (41%) were not sure if the feedback process concludes with a discussion for developing a plan of action for improvement.
- ***Tutor performance:*** Students mean rating for their tutors’ performance ranged from 3.64 (focusing on specific and relevant performance) to 3.80/5 (using of clear and relevant language) (Table-I). Sixty one percent of the students agreed / strongly agreed that their tutors judged their performance rather than personality, 136 (65%) agreed/strongly agreed that their tutors use clear and relevant language, and 132 (63%) agreed/strongly agreed that tutors give them a chance to discuss the feedback comments. More first year students agreed / strongly agreed that their tutor uses clear and relevant language and gives them a chance to discuss the feedback comments (p=0.001 and p=0.002, respectively).
- ***The content of the PBL feedback:*** The students’ mean total score for feedback content was 3.62/5.0 for knowledge acquisition and cognitive skills and 3.67/5.0 for interaction and participation in group function. A larger proportion of first year students significantly agreed/strongly agreed on the knowledge acquisition and participation in group function contents of the PBL feedback compared to second-year students (p=0.021 and p=0.012, respectively). (Table-I)

**Table-I:** Students Perceptions of the Process of the PBL Feedback*

Questions	Mean ±SD	Categories	All N=209 n (%)	First Year N=110 n (%)	Second Year N=99 n (%)	p-value (Year levels)
*Process of feedback provision*						
*Steps of feedback that the tutor follows*						
Asks you to assess your own performance	3.63 ± 1.05	Agree	124 (59%)	70 (64%)	54 (55%)
		NS	60 (29%)	29 (26. %)	31 (31%)
		Disagree	25 (12%)	11 (10 %)	14 (14%)
Tells you what went well	3.63 ± 1.17	Agree	128 (61%)	72 (66%)	56 (57%)	0.180
		NS	50 (24%)	22 (20%)	28 (28%)
		Disagree	31 (15%)	16 (15%)	15 (15 %)
Tells you areas for improvement	3.67 ±1.15	Agree	127 (61%)	73 (66%)	54 (55%)	0.229
		NS	51 (24%)	21 (19%)	30 (30%)
		Disagree	31 (15%)	16 (15%)	15 (15%)
Develop with you a plan of action for improvement	3.56 ± 1.06	Agree	108 (52%)	57 (52%)	51 (52%)	0.974
		NS	85 (41%)	44 (40%)	41 (41%)
		Disagree	16 (8%)	09 (8%)	07 (7%)
*Tutor performance*							
Judges performance rather than personality	3.71 ± 1.11	Agree	128 (61%)	75 (69%)	52 (53%)	0.163
		NS	54 (26%)	21 (19%)	33 (33%)
		Disagree	27 (13%)	13 (12%)	14 (14%)
Focuses on a specific and relevant performance	3.64 ± 1.01	Agree	122 (58%)	72 (65%)	50 (51%)	0.257
		NS	64 (31%)	27 (25%)	37 (37%)
		Disagree	23 (11%)	11 (10%)	12 (12%)
Uses clear and relevant language	3.80 ± 1.03	Agree	136 (65%)	83 (75%)	53 (53%)	0.001
		NS	54 (26%)	15 (14%)	39 (39%)
		Disagree	19 (9%)	12 (11%)	07 (7%)
Gives chance for you to discuss the feedback comments	3.73 ± 1.05	Agree	132 (63%)	80 (73%)	52 (53%)	0.002
		NS	55 (26%)	16 (15%)	39 (39%)
		Disagree	22 (11%)	14 (13%)	08 (8%)
Content of feedback						
Knowledge acquisition and cognitive skills	3.62 ± 0.99	Agree	119 (57%)	71 (65%)	48 (49%)	0.021
		NS	68 (33%)	25 (23%)	43 (43%)
		Disagree	22 (11%)	14 (13%)	08 (8%)
Interaction and participation in group function	3.67 ± 1.00	Agree	123 (59%)	75 (78%)	48 (49%)	0.012
		NS	66 (32%)	23 (21%)	43 (43%)
		Disagree	20 (10%)	12 (11%)	08 (8%)

Note: Agree- agree/strongly agree; NS-not sure; Disagree-disagree/strongly disagree, * Percentages are rounded to the nearest whole number.

### 2. Benefits of the provided feedback

The agreement rates regarding the helpfulness of PBL feedback is shown in Table-II. More first year students agreed/strongly agreed that the provided feedback helped them to improve their knowledge acquisition (p=0.003), problem-solving (p=0.004), communication (p=0.002), teamwork (p<0.001), time management (p<0.001) and self-assessment skills (p=0.001) compared to second year students.

**Table-II:** Students perceptions of the Benefits of the PBL Feedback*

Items	Mean ±SD	Categories	All N=209 n (%)	First Year N=110 n (%)	Second Year N=99 n (%)	p-value (Year levels)
*A. In improving skills:*						
Knowledge acquisition	3.66 ± 1.04	Agree	121 (58%)	74 (67%)	47 (48%)	0.003
		NS	65 (31%)	21 (19%)	44 (44%)	
		Disagree	23 (11%)	15 (14%)	08 (8%)	
Problem-solving	3.67 ± 1.03	Agree	125 (60%)	74 (67%)	51 (52%)	0.004
		NS	61 (29%)	20 (18%)	41 (41%)	
		Disagree	23 (11%)	16 (15%)	07 (7%)	
Communication	3.73 ± (0.99)	Agree	127 (61%)	79 (72%)	48 (49%)	0.002
		NS	64 (31%)	20 (18%)	44 (44%)	
		Disagree	18 (9%)	11 (10%)	07 (7%)	
Teamwork	3.68 ± 1.03	Agree	125 (60%)	79 (72%)	46 (47%)	<0.001
		NS	59 (28%)	15 (14%)	44 (44%)	
		Disagree	25 (12%)	16 (15%)	09 (9%)	
Time management	3.64 ± 1.06	Agree	121 (58%)	78 (71%)	43 (44%)	<0.001
		NS	61 (29%)	18 (16%)	43 (43%)	
		Disagree	27 (13%)	14 (13%)	13 (13%)	
Self-assessment	3.67 ± 1.06	Agree	122 (58%)	76 (69%)	46 (47%)	0.001
		NS	64 (31%)	20 (18%)	44 (44%)	
		Disagree	23 (11%)	14 (13%)	09 (9%)	
*B. In appreciating value of:*								
Receiving regular feedback on performance	3.61 ± 1.08	Agree	113 (54%)	70 (64%)	43 (43%)	<0.001
		NS	70 (34%)	24 (22%)	46 (47%)	
		Disagree	26 (12%)	16 (15%)	10 (10%)	
Continuous performance improvement	3.45 ± 1.00	Agree	92 (44%)	53 (48%)	39 (39%)	0.033
		NS	96 (46%)	43 (39%)	30 (30%)	
		Disagree	21 (10%)	14 (13%)	07 (7%)	

*Note:* Agree- agree/strongly agree; NS-not sure; Disagree-disagree/strongly disagree, * Percentages are rounded to the nearest whole number

## DISCUSSION

Timely feedback allows students to self-assess their performance, and allows their tutor to guide them on how to improve their performance.^5^,^6^ In the current study, about half of the students’ agreed/strongly agreed that feedback provision regularly followed the frequency and timing planned by the college, and the agreement was slightly higher for individual feedbacks provided at the middle of the block. Aldrees et al.^7^ reported in 2015 that 55.3% of medical students agreed about feedback regularity. Both our study and theirs showed no statistically significant differences across the year of study. A probable explanation might be the presence of variable practices by the different tutors. Motivating the students to be pro-active feedback seekers can tremendously influence the regularity and timeliness of feedback provision.^2^

Many socio-cultural factors influence students’ satisfaction with feedback. In the United Kingdom, a high percentage of students’ dissatisfaction with the feedback provided was encountered in some institutions despite the timely, extensive feedback received.^8^ A supportive learning environment translates to a decrease in stress and anxiety and is directly related to learning enhancement.^9^ A positive finding of the current study is that the majority of our students perceived their PBL feedback provision environment as relaxed and non-threatening, with no significant reported year level differences. The current study results are higher than the 40-50% results of Al-Ayed & Sheikh.^10^ Our results indicate a shift from the traditional one-way feedback provision to a more dialogic bidirectional ATA approach by about half of our PBL tutors. French et al.^4^ state that the ATA model has the advantage of being a “reinforcing and modifying feedback” approach.

Our students reported a positive impact of the feedback they received on their learning (mean summative agreement scores ranged from 3.45 to 3.73). About 60% agreed on the helpfulness of the PBL feedback in improving their non-technical skills. Non-technical skills as communication, professionalism, and teamwork are among the challenging areas for medical educators.^11^ Our College careful planning for integrating these skills into the students learning experience is evident. The outcomes of these efforts can be maximized by applying more quality assurance methods. The variability in the frequency, timing, quality and outcomes of the PBL feedback provision reported in this study requires investigation. A collaboration of the PBL, Curriculum, and Academic Quality Units, to plan for peer-reviewing of feedback provision in the different classes, is recommended. Students should also be aware of their active role in the initiation and implementation of a successful feedback process. PBL facilitator’s training workshops are to continue to emphasize the bidirectional, dialogic ATA approach.

### Limitations of the study

To explore non-response as a source of bias, we invited a random sample of 20 non-responding students to respond to the survey. No statistically significant differences were found between their responses and those collected during the original data collection time. To enhance the response rates and gather regular monitoring data, we recommend that the evaluation of feedback be added to the Academic Quality Unit block survey. Further studies are expected to expand on the findings of this descriptive study and investigate the variations in different tutors’ practices based on gender, attendance of faculty development workshops, academic qualifications, and perceptions of the value of PBL feedback.

## CONCLUSION

Many of our PBL tutors have started the shifting from the traditional one-way PBL feedback provision into a more dialogic bidirectional approach. Feedback process, quality and benefits are well perceived by more than half of our respondents. Continuing the faculty development efforts and peer-reviewing of the PBL feedback process and outcomes, and periodic students’ satisfaction surveys are recommended.

### Authors’ Contribution:

**MS:** Study design, survey design, data collection and manuscript writing. **AC. I & SA. K:** Data collection, statistical analysis, manuscript writing, and revision. **NK:** Survey design, manuscript editing and review.

## References

[1] Telio S, Ajjawi R, Regehr G (2015). The “educational alliance”as a framework for reconceptualizing feedback in medical education. Acad Med.

[2] Ramani S, Könings KD, Ginsburg S, van der Vleuten CPM (2019). Twelve tips to promote a feedback culture with a growth mind-set:Swinging the feedback pendulum from recipes to relationships. Med Teach.

[3] Webb A, Moallem M (2016). Feedback and Feed-Forward for Promoting Problem-Based Learning in Online Learning Environments. Malays J Learn Instr.

[4] French JC, Colbert CY, Pien LC, Dannefer EF, Taylor CA (2015). Targeted Feedback in the Milestones Era:Utilization of the Ask-Tell-Ask Feedback Model to Promote Reflection and Self-Assessment. J Surg Educ.

[5] Tanes Z, Arnold KE, King AS, Remnet MA (2011). Using Signals for appropriate feedback:Perceptions and practices. Comput Educ.

[6] Lefroy J, Watling C, Teunissen PW, Brand P (2015). Guidelines:the do's, don'ts and don't knows of feedback for clinical education. Perspect Med Educ.

[7] Al-Drees MA, Khalil MS, Irshad M, Abdulghani HM (2015). Students'perception towards the problem-based learning tutorial session in a system-based hybrid curriculum. Saudi Med J.

[8] Robinson S, Pope D, Holyoak L (2013). Can we meet their expectations?Experiences and perceptions of feedback in first year undergraduate students. Assess Eval High Educ.

[9] Moscaritolo LM (2009). Interventional strategies to decrease nursing student anxiety in the clinical learning environment. J Nurs Educ.

[10] Al-Ayed IH, Sheik SA (2008). Assessment of the educational environment at the College of Medicine of King Saud University, Riyadh. East Mediterr Health J.

[11] Damewood RB, Blair PG, Park YS, Lupi LK, Newman RW, Sachdeva AK (2017). Taking Training to the Next Level:The American College of Surgeons Committee on Residency Training Survey. J Surg Educ.

